# Bio-Enhanced Degradation Strategies for Fluoroquinolones in the Sewage Sludge Composting Stage: Molecular Modification and Resistance Gene Regulation

**DOI:** 10.3390/ijerph19137766

**Published:** 2022-06-24

**Authors:** Xingyan Jin, Yuanyuan Zhao, Zhixing Ren, Panpan Wang, Yu Li

**Affiliations:** 1 School of Environment, Harbin Institute of Technology, Harbin 150006, China; 21s029010@stu.hit.edu.cn; 2 MOE Key Laboratory of Resources and Environmental Systems Optimization, North China Electric Power University, Beijing 102206, China; zyy0210@ncepu.edu.cn; 3 College of Forestry, Northeast Forestry University, Harbin 150040, China; renzhixingryy@outlook.com

**Keywords:** fluoroquinolones, sewage sludge composting, biodegradation, 3D-QSAR, MD simulation, resistance genes

## Abstract

The molecular/protein–protein docking and the index normalization method assisted by the entropy weight method were used to quantitatively evaluate the biodegradability of fluoroquinolones (FQs) under different biodegradation systems. Four biodegradability three-dimensional quantitative structure–activity relationship (3D-QSAR) models of FQs were constructed to design FQ derivatives with improved biodegradability. Through the evaluation of the environmental friendliness and functional properties, the FQ derivatives with high biodegradability, improved functionality, and environmental friendliness were screened. Moreover, four bio-enhanced degradation scenarios of FQs were set up according to the different temperatures and carbon–nitrogen ratio (C/N) in the sewage sludge composting stage, and the molecular dynamic (MD) simulation assisted by protein–protein docking was used to screen the external environmental factors that promote the degradation of FQs by thermophilic bacteria or group under different scenarios. Finally, MD simulation assisted by sampling method was used to validate and screen the application scheme of field measures to enhance the expression of antibacterial resistance of FQ derivatives in an agricultural soil environment after activated sludge land use. This study aims to provide theoretical support for the development of highly biodegradable FQ derivatives and the mitigation of potential risks that FQs may pose to the environment and humans through the food chain.

## 1. Introduction

Fluoroquinolones (FQs), with better broad-spectrum and pharmacokinetics than the first generation of quinolones antibiotics, developed rapidly from 1970 to 1980 [1]. FQs are a class of synthetic broad-spectrum antibacterial agents, they are widely used in clinical treatment in hospitals and veterinary practices [2]. However, only a small fraction of FQs are absorbed by the organism. Up to 90% of oral doses of drugs are discharged in the form of original drugs or metabolites through feces and urine [3,4], and then transferred to wastewater treatment plants by urban sewage pipes for centralized treatment [5]. Current control indexes of sewage treatment plants do not include antibiotics [6]; thus, antibiotics cannot be effectively degraded in sewage treatment plants. After treatment, the effluent still has residual active ingredients, which are absorbed into the environmental medium [7]. Therefore, it is particularly important to seek FQ substitutes with enhanced microbial degradability and screen suitable external environmental conditions for degradation.

In recent years, researchers at home and abroad have begun to study the effect of FQ biodegradation in high-temperature aerobic compost. Jiang et al. explored the degradation mechanism of FQs in compost at 55 °C, and conducted experiments with Norfloxacin (NOR) and ofloxacin (OFL). The study proved that the main removal pathway of FQs was biodegradation, the NOR removal rate was 31.37%, and the OFL removal rate was 26.36% [8]. Selvam et al. found that under the condition of high-temperature composting, the Ciprofloxacin (CIP) removal rate could reach 82.9% at the initial concentration of 10 mg/kg [9]. Meng pointed out that the longer the duration of the high temperature stage of aerobic composting, the more conducive to the degradation and removal of pollutants in the heap, and thermophilic bacteria were the dominant species to degrade pollutants [10]. Five FQ compounds: NOR, CIP, Lomefloxacin (LOM), Enrofloxacin (ENR), and Salfloxacin (SAR) were composted at high temperature and added with high-temperature-resistant strains. Under the above circumstances, the removal effect of FQs with different concentrations in the composting process and the influence of exogenous heat-resistant strains on the removal of FQ compounds were studied. It was found that high temperature composting on 42 days could remove 48.4~77.1% of FQs in chicken manure. In addition, the removal rate of NOR and LOM was significantly improved after the addition of high-temperature resistant strains. According to colony morphology and microscope observation, the strain was preliminarily identified as *Fibroomonas flavanum*. Pan et al. isolated a thermophilic bacterium that could degrade CIP from the sludge in an antibiotic pharmaceutical factory [11]. According to the 16S rRNA gene sequence analysis, it was found that the strain was closely related to thermophilic bacteria, named C419, and the optimal temperature of CIP degradation was 70 °C. In addition, strain C419 also degraded other fluoroquinolones, including OFL, NOR, and ENR, suggesting that C419 strain may be a new helper bacterial resource for biodegrading fluoroquinolone residues in thermal environments. Tumini et al. used *Bacillus licheniformis* to test FQs in milk, to indicate the interaction between them, and *Bacillus licheniformis* was also a thermophilic bacterium [12]. Therefore, the biodegradation of FQs by thermophilic bacteria has attracted extensive attention.

With the widespread use of antibiotics, the drug resistance of antibiotic-resistant bacteria (ARB) caused by antibiotic resistance genes (ARGs) has aroused widespread concern in society [13]. According to the 2017 monitoring data in the Status Report of Antimicrobial Management and Bacterial Resistance in China [14], the average isolation rate of quinolone-resistant *Escherichia coli* in China was 51.0%, indicating a prominent problem of bacterial drug resistance. FQs molecules produce bactericidal effects by binding with DNA cyclase in the recipient bacteria and obstructing its asexual reproduction process [15]. However, due to the expression of ARGs, the recipient bacteria will express the resistant DNA gyrase mutant protein, thus weakening the bactericidal activity of FQs [16]. Ren et al. constructed 29 kinds of DNA helicase mutant proteins of *Escherichia coli* resistant to FQs through a homologous modeling algorithm, and the drug resistance was 25.98% higher than before mutation [17]. Therefore, enhancing the binding ability of FQ derivative molecules to drug-resistant DNA helicase mutant proteins is also one of the main objectives of the research. Wastewater treatment plants need to deal with a large amount of sewage every day, including domestic sewage, medical wastewater, aquaculture wastewater, and other sewage [18]. Among them, a large number of ARBs and ARGs enter the treatment system of wastewater treatment plants along with all kinds of wastewater [19]. After being treated by the wastewater treatment plant, the activated sludge, as the end product, eventually becomes the enrichment pool of ARB and ARGs in the sewage treatment system [20]. Land use of activated sludge is considered to be one of the most promising disposal methods for sludge [21]. However, with the promotion of land use of activated sludge, the incompletely treated pollutants (such as ARB and ARGs) in activated sludge will be transferred to the soil environment, even with appropriate pre-treatment [22]. Therefore, it is of far-reaching significance to study the inhibition of bacterial resistance expression in soil environments.

The purpose of this study was to explore the enhanced biodegradability of FQs in the sludge aerobic composting process and to design FQ derivative molecules to screen the easily biodegradable molecular replacement of FQs. It provided theoretical guidance for reducing the adverse effects of FQs discharged into the environment, and it was of great significance for optimizing the degradation of aerobic sludge. Firstly, the molecular docking was used to characterize the biodegradabilities of 49 FQs by thermophilic bacteria *Thermus* sp. C419, *Bacillus licheniformis*, and *Cellulomonas flavigena* (the degraded proteins were represented by 1GKQ, 1OB0, and 5M0K, respectively). Secondly, the entropy weight method and index normalization method were used to integrate the degradation effect of three thermophilic bacteria on 49 FQs compounds. The concept of comprehensive biodegradability of FQs was proposed for the first time, and the comprehensive biodegradability of FQs was predicted by thermophiles. In addition, a 3D-QSAR model based on the biodegradability evaluation index of FQs (hereinafter referred to as biodegradability 3D-QSAR model) was constructed. According to the 3D isopotential diagram of the biodegradability 3D-QSAR model, the substitution sites of FQs molecules were screened. Moxifloxacin (MOX) was used as the target molecule to design FQ derivatives with high biodegradability. Finally, protein–protein docking assisted molecular dynamic (MD) simulation was used to screen regulatory schemes that could promote the degradation of FQs by combined thermophilic bacteria and enhance the expression of anti-bacterial resistance of MOX derivatives. This paper aims to reduce the impact of residual FQs on the ecological environment and human health and has important practical significance for protecting the ecological balance and achieve green and sustainable development.

## 2. Materials and Methods

### 2.1. Source of FQs and Biodegradation Enzymes in Sewage Sludge Composting Stage

The residual problem of FQs has harmed aquatic and soil organisms. For example, a high concentration of enrofloxacin (ENR) inhibits the growth of radish and other plants and also leads to the generation of drug-resistant bacteria and resistance genes [23]. It is worth noting that FQs can be removed by thermophilic bacteria at high-temperature composting. Therefore, considering the usage of FQs and their possible adverse effects on the environment and human health, 49 kinds of FQs, such as difloxacin (DIF), gatifloxacin (GAT), and orbifloxacin (ORB), were selected as research objects in this paper to study the biodegradability of their sewage sludge composting stage.

The target receptors in this paper are derived from three kinds of thermophilic bacteria: *Thermus* sp. C419 belongs to extreme thermophilic bacteria and is a kind of aerobic bacteria. The sewage sludge contains protein 1GKP that can degrade FQs. The other is *Bacillus licheniformis*, which is an aerobic and heat-resistant bacterium. It has been proved that this bacterium can detect FQs drugs in milk. Among them, 1BLI (α amylase)/1VJS (α amylase precursor) plays a major role. The third is *Cellulomonas flavigena*, which is a facultative anaerobe and a heat-resistant bacterium. It can degrade FQs and contains 5M0K (β-xylanase: hydrolase) protein. Therefore, in this paper, *Thermus* sp., *Bacillus Licheniformis*, and *Cellulomonas flavigena* were selected as target receptors to study the single and combined biodegradability of FQs in the sewage sludge composting stage. The above three target receptor structures were obtained from the PDB (https://www.rcsb.org/, accessed on 11 August 2021) database, and their PDB IDs were 1GKQ, 1OB0, and 5M0K, respectively.

### 2.2. Characterization of FQs Molecular Biodegradability in Sewage Sludge Composting Stage—Molecular Docking Method

It is known that the biodegradability of FQs is related to the interaction between *Thermus* sp., *Bacillus Licheniformis*, and *Cellulomonas flavigena*, and the docking score between ligand and receptor can be used to characterize the biodegradability [24]. Therefore, 49 kinds of FQs and three biodegradable target receptors were loaded into Discovery Studio 2020 (DS 2020) software (BIOVIATM, Vélizy-Villacoublay, France) in this paper. The LibDock module was used to define the three selected proteins (1GKQ, 1OB0, and 5M0K) as receptor molecules and FQs as ligand molecules, respectively. The Find Sites from the Receptor Cavities under the Define module was used to search for the active site that may be bound to the Receptor. Finally, the ligand molecule was integrated into the formed active cavity for rapid docking with the receptor protein. During the Docking process, User Specified was selected, Max Hit to Save was set to 10, Conformation Method was set to BEST, and other default settings were set. Finally, the LibDock score was used to analyze the strength of ligand–receptor interaction. The higher the score, the stronger the ligand–receptor interaction was, indicating the stronger the ability of thermophiles to degrade FQs, and vice versa.

### 2.3. Characterization of the Combined Biodegradability of Thermophilic Bacteria of FQs Sewage Sludge Composting Stage—Entropy Weight Method and Index Normalization Method

FQs can be effectively degraded by 1GKQ, 1OB0, and 5M0K in the sewage sludge composting stage. However, the process of evaluating the combined biodegradability of FQs molecules by thermophiles is complicated and fuzzy, and it is impossible to obtain the results by very accurate evaluation methods. The combined use of the entropy weight method and index normalization method can solve the problem of incomplete data and fuzzy factors in some cases [25]. Therefore, this paper attempts to comprehensively evaluate the combined biodegradability of the thermophilic bacteria community of FQs in the sewage sludge composting stage by using the entropy weight method and index normalization method.

First, the entropy weight method was used to calculate the weight of the effects of 1GKQ, 1OB0, and 5M0K receptors on the biodegradability of FQs. In the first step, the influence specific gravity *P_ij_* of the *j*th receptor on the degradation of the *i*th FQs was calculated. The second step was to calculate the entropy *e_j_* of the *j*th protein. Thirdly, the entropy weight *ω_j_* of the *j*th protein was calculated, and the *ω* = {*ω*_1_, *ω*_2_, *ω*_3_} was obtained. The higher the *ω_j_* value was, the greater the effect of the *j*th protein on the biodegradation of FQs was. Secondly, the range method was used to perform dimensionless normalization on the degradability of the *j*th protein to the *i*th FQs. The degradation values of the *j*th protein to 49 kinds of FQs were sequenced from small to large, and the minimum and maximum values were *v_j_*_min_ and *v_j_*_max_, respectively. In this paper, the degradability of FQs by thermophilic bacteria was expected to be improved. Therefore, the parameters were defined as positive indicators, with *v_j_*_min_ and *v_j_*_max_ assigned as 9 and 1. Then, according to Equation (4), the values between the two were proportionally corresponding to the values between 1 and 9. Finally, the comprehensive value b_i_ of the degradation of the *i*th FQs by three kinds of thermophilic bacteria was calculated. The specific process is shown in Equations (1)–(5).

(1) $$P_{ij}=v_{ij}/\overset{m}{\underset{i}{\sum }}v_{ij}$$

(2) $$e_{j}=-\frac{1}{\ln m}\sum_{i=1}^{m}p_{ji}\ln p_{ji}$$

(3) $$\omega_{j}=(1-e_{j})/\sum_{j=1}^{n}\left(1-e_{j}\right)$$

(4) $$a_{ji}=1+(v_{ji}-v_{j\min})\times 8/(v_{j\max}-v_{j\min})$$

(5) $$b_{i}=\sum_{j=1}^{3}\left(\omega_{j}\times a_{ji}\right)$$

where, *i* = 1, …, 49 represent 49 kinds of FQs molecules respectively; *j* = 1, 2, 3 represent three proteins, 1GKQ, 1OB0, and 5M0K, respectively; *P_ij_* represents the influence specific gravity of the *j*th receptor on the degradation of the *i*th FQs; *e_j_* represents the entropy of the *j*th protein; *ω_j_* is the entropy weight of the *j*th protein; *v_j_*_min_ and *v_j_*_max_ represent the minimum and maximum values were, respectively; *v_ij_* represents the biodegradability parameter of the *j*th protein to the *i*th FQs; *b_i_* represents the comprehensive value of the degradation of the *i*th FQs by three kinds of thermophilic bacteria.

### 2.4. Construction of 3D-QSAR Models of FQs Biodegradability for Single and Combined Thermophilic Bacteria—CoMFA Method

In this paper, SYBYL-X 2.0 software (Tripos Assoc., St Louis, MO, USA) from the company Tripos was used for 3D-QSAR analysis. In the combined thermophilic bacterial degradation model, the combined biodegradation value of 30 FQs molecules was selected as the dependent variable and the molecular structure parameters of FQs were used as the independent variable. Among them, data sources of 23 and 8 FQs molecules were selected as training set and test set, respectively (template molecule existed in both training set and test set). In the 3D-QSAR model of 1GKQ biodegradation of FQs, the biodegradation values of 16 FQs molecules were selected as dependent variables and the molecular structure parameters of FQs were used as independent variables. Data sources of 12 and 5 FQs molecules were selected as training set and test set, respectively (template molecules existed in both training set and test set). In the 3D-QSAR model for 1OB0 biodegradation of the FQ molecule, the biodegradation values of 23 FQs molecules were selected as dependent variables and the molecular structure parameters of FQs were used as independent variables. Data sources of 18 and 6 FQs molecules were selected as training set and test set, respectively (template molecules existed in both training set and test set). In the 3D-QSAR model of 5M0K biodegradation of FQs, biodegradation values of 31 FQs molecules were selected as dependent variables and molecular structure parameters of FQs were used as independent variables. Data sources of 24 and 8 FQs molecules were selected as training set and test set, respectively (template molecules existed in both training set and test set).

**Figure 1:**
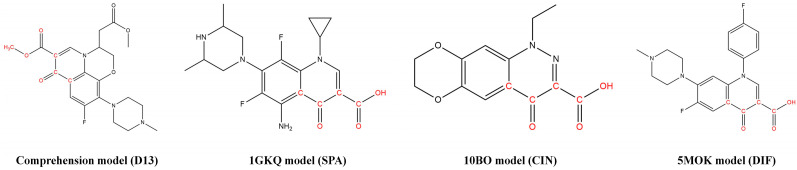
Schematic diagram of template molecules and their common skeleton in FQ biodegradability models.

In the process of the 3D-QSAR model construction, the FQs molecular structure directly drawn in SYBYL-X 2.0 software is not the most stable conformation. In the case of unknown receptors, the conformation of the lowest energy state of the molecular structure was generally selected as the active conformation. With the Minimize module in SYBYL-X 2.0 software and Powell conjugate gradient method, the Tripos force field was selected and a Gasteiger–Huckel charge was added. The energy convergence is limited to 0.005 kJ/mol for 10,000 iterations, and the other values were the default. In the case of unknown receptors, compounds with the most ideal activity value are usually selected as template molecules. In this paper, D13, SPA, CIN, and DIF were selected as template molecules in the combined biodegradation model, 1GKQ degradation model, 1OB0 degradation model, and 5M0K degradation model. The red area in Figure 1 was used as the common skeleton to perform molecular superposition of FQs through the Align Database module [26].

When calculating the internal verification parameters of the 3D-QSAR model, the molecular field type was electrostatic field (E) and steric field (S), and the dielectric constant was related to the distance, and the threshold was 125.4 kJ/mol. The default values of other parameters were used to calculate and obtain the molecular field parameters. The combined biodegradation value, 1GKQ degradation value, 1OB0 degradation value, and 5M0K degradation value of FQs were imported into the Training table, and the evaluation parameters of four groups of 3D-QSAR models were automatically calculated by Sybyl-X 2.0 through Autofill. Partial least squares (PLS) analysis was used to establish the relationship between the structure and bioactivity of target compounds. When using PLS analysis, firstly, the leave-one-out method was used to cross-verify the compounds in the training set, and the cross-validation coefficient *q*^2^ and the optimal principal component number *n* were obtained. Secondly, regression analysis was carried out through No Validation regression, and the non-cross-validation coefficient *r*^2^ was obtained to complete internal validation of the 3D-QSAR model. Finally, the $r_{\mathrm{pred}}^{2}$, calculated by Equation (6) and which met the requirements of the model, was calculated to complete the external validation of the 3D-QSAR model, thus completing the construction of the COMFA model [27].

(6) $$r_{\mathrm{pred}}^{2}=1-\sum {\left(y_{i}-{\hat{y}}_{i}\right)}^{2}/\sum {\left(y_{i}-{\bar{y}}_{\mathrm{EXT}}\right)}^{2}$$

### 2.5. Optimization of External Environmental Conditions for Combined Biodegradability Improvement of FQs Sewage Sludge Composting Stage under Multiple Scenarios

#### 2.5.1. Characterization of Key Proteins Affecting the Combined Biodegradability of FQs Sewage Sludge Composting Stage—Protein–Protein Docking

To simulate the combined biodegradation system of FQs by thermophilic bacteria in the sewage sludge composting stage, this paper selected the complex of three target proteins (1GKQ&1OB0&5M0K) as the research object and realized the docking between proteins with the help of ZDOCK and RDOCK modules in DS 2020 software. Firstly, the three target protein structures were loaded into DS 2020 software, and the Dock and Analyze Protein Complexes modules under Macromolecules were used to preprocess the target proteins. Secondly, the optimal binding configuration of the protein–protein complex was found by using the ZDOCK module and optimized under the RDOCK module. During docking, 1GKQ and 1GKQ&1OB0 complexes were defined as receptor molecules, and 1OB0 and 5M0K were defined as ligand molecules, to which the binding of the target protein to the target protein was carried out accordingly. Finally, the obtained target protein complex was used to characterize the combined biodegradation system of thermophilic bacteria in the sewage sludge composting stage of FQs [28].

#### 2.5.2. Screening of External Environmental Conditions Affecting the Combined Biodegradability of FQs Sewage Sludge Composting Stage and Setting of Degradation Scenarios

Sewage sludge composting is an effective way to realize the reduction, resource recovery, and harmless treatment of organic waste [29]. At present, the main factors affecting sewage sludge composting include temperature, C/N ratio, organic matter (OM), phosphoric organic substances (POS), additives, etc. Reasonable regulation of these parameters in the process of sewage sludge composting is conducive to composting and can improve the quality of composting [30]. Studies have found that temperature change during composting can reflect the activity of microorganisms in composting materials well, and is an important indicator of organic degradation rate and harmless composting [31]. At the same time, when the initial C/N ratio of composting materials is 25–35, it can not only reduce nitrogen loss during composting but also speed up the composting process [32]. In addition, appropriate exogenous additives can effectively reduce ammonia volatilization and greenhouse gas emission in the composting process, and promote microbial degradation of organic pollutants [33]. The exogenous additives mainly considered in this paper include wood vinegar (WV), citric acid (CA), apple pomace (AP), urethane residue (UR), nitrilotriacetic acid (NTA), and medical stone (MS).

Firstly, four biodegradation scenarios were set according to composting temperature and C/N ratio. In scenario 1, the composting temperature was 35 °C and C/N was 25. In scenario 2, the composting temperature was 35 °C and C/N was 35. In scenario 3, the composting temperature was 55 °C and C/N was 25. In scenario 4, the composting temperature was 55 °C and C/N was 35. Secondly, MD simulation technology was used to add OM, POS, and six exogenous additives into the compound system of FQ derivatives (Derivative-10 as an example) and 1GKQ&1OB0&5M0K, respectively. Finally, each biodegradation scenario included a blank group and eight experimental groups, and a total of 36 combination schemes are used for subsequent MD simulation. The specific schemes are shown in Table 1 (0 represents no addition and 1 represents addition).

#### 2.5.3. Optimization of External Environmental Conditions for Combined Biodegradability Improvement of FQ Sewage Sludge Composting Stage—MD Simulation

The MD simulations process was based on the pdb2gmx function in the GROMACS4.6.7 software (Berendsen Laboratory, Göttingen University, Göttingen, Germany), using the Gromos96 54A7 force field to create the topologies of the protein. The automatic topology generator (ATB) was used to create the ligand molecule topologies. The complex system of Derivative-10 and 1GKQ&1OB0&5M0K receptor was placed in a cubic box with the distance between the box boundary and the complex of 1.0 nm. The SPC-type water model (SPC216) was then added to the box. Before the simulation starts, an appropriate amount of sodium ion and chloride ion to the box for charge balance should be added to make the whole system electrically neutral. The whole simulation process was divided into four steps, namely energy minimization, NVT temperature control, NPT pressure control, and MD balance simulations. The steepest descent method was used for energy minimization, and the V-rescale heat bath method was used to control the temperature (i.e., T = 308 K or 328 K) of the system during the simulation process. The Parrinello–Rahman pressure bath method was used to control the constant pressure conditions of the system during the simulation process (P = 0.1 MPa). During MD simulations, the position constraints were removed, and the real complex in vivo environment was simulated using the leapfrog Newton integration method. Specifically, the simulation step size was 2 fs, and the number of simulation steps was 100,000 steps. The particle-mesh Ewald method was used to calculate the long-range electrostatic interactions, and the van der Waals interactions were calculated by using the truncation method (the truncation radius is 1.4 nm).

The objective of this study was to optimize the external environmental factors of thermophilic bacteria community combined biodegradability of Derivative-10 under four biodegradation scenarios in sewage sludge composting stage, simulation of blank groups, and experimental groups were set up. The blank groups were not added POS, OM, and exogenous additives. The experimental groups were added based on Table 1. The binding of Derivative-10 with 1GKQ&1OB0&5M0K complex receptor was simulated. The molecular Mechanics/Poisson–Boltzmann Surface Area (MMPBSA) method was used to calculate the binding energy and determine whether external environmental conditions could improve the combined biodegradability of thermophilic bacteria of FQs in the sewage sludge composting stage. Finally, the optimal combination scheme was determined [34].

When MMPBSA is used to calculate the binding free energy, it is necessary to sample the equilibrium trajectory of protein and ligand complex and calculate the binding free energy of the complex, protein, and ligand, respectively. MMPBSA is an endpoint method, which involves calculating the difference of free energy between two states. The binding free energy calculation formula is:

(7) $$G_{bind}=G_{complex}-G_{free-protein}-G_{free-ligand}$$

In solution, the binding energy of molecules:

(8) $$G=E_{gas}-TS_{gas}+G_{solvation}$$

where the solvation free energy can be decomposed into polar and non-polar parts:

(9) $$G_{solvation}=G_{polar}+G_{nonpolar}$$

## 3. Results and Discussion

### 3.1. Quantitative Analysis of Combined Biodegradation Levels of Single Thermophilic Bacteria and Thermophilic Group to FQs in Sewage Sludge Composting Stage

It is of great practical importance to quantify the biodegradation level of FQs in the sewage sludge composting stage for the degradation effect of different proteins on FQs. In this study, 49 FQs were selected for molecular docking with target receptors in three thermophilic bacteria, and quantified by characterizing the single biodegradability of the target receptors to FQs using the docking scores (Libdock Scores) (Table 2).

In addition to quantifying the single biodegradability of 49 FQs using three proteins, 1GKQ, 1OB0, and 5M0K, this study calculated and characterized the combined biodegradability of 49 FQs in the sewage sludge composting stage based on the entropy weight method and index normalization method introduced in Section 2.3, and the specific results are shown in Table 3.

### 3.2. Construction and Evaluation of the Biodegradability 3D-QSAR Models of FQs by Thermophilic Bacteria in Sewage Sludge Composting Stage

The evaluation results for the biodegradability 3D-QSAR models of FQs by thermophilic bacteria in the sewage sludge composting stage were calculated. For internal validation, the optimal number of components (*n*) of the 3D-QSAR models were 10, 4, 5, and 10 and the cross-validated values (*q*^2^) were 0.823, 0.765, 0.738, and 0.774 in the comprehension, 1GKQ, 1OB0, and 5M0K biodegradation models, respectively. When the value of *q*^2^ was greater than 0.5, the model had a good prediction ability. Moreover, the coefficients of determination (*r*^2^) (i.e., 1.000, 0.984, 0.994, and 1.000) of the four models were all greater than 0.8, indicating that the models had a good fitting ability. Additionally, the (*r*^2^ *− q*^2^*)/r*^2^ were 17.70%, 22.26%, 25.75%, and 22.60% (i.e., <30%), respectively, which suggested that the models were not overfitted. For the external validation, the established comprehension, 1GKQ, 1OB0, and 5M0K biodegradation 3D-QSAR models were used to predict the biodegradability of FQs in the test sets to verify the accuracy of the obtained models. The external validation parameters were calculated by Equation (6). The interaction test coefficients ($r_{\mathrm{pred}}^{2}$) of the four 3D-QSAR models (0.711, 0.984, 0.770, and 0.883) were all greater than 0.6. These results suggested that the models had good external prediction ability and thus can be used to accurately predict the biodegradability of FQs in the sewage sludge composting stage.

### 3.3. Molecular Modification of FQs with Improved Biodegradation and Prediction of Relevant Properties for FQ Derivatives

#### 3.3.1. Determination of Modified Sites and Groups for FQs with High Combined Biodegradability Based on the Contour Maps

**Figure 2:**
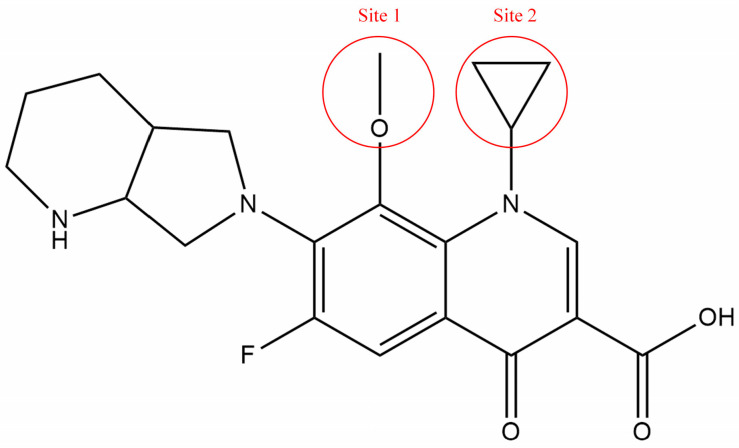
The molecular structure and the modified sites of MOX.

In this study, moxifloxacin (MOX), a widely used FQ, was selected as the modified compounds, and two groups (1-position substituent, C_3_H_5_; 2-position substituent, OCH_3_) attached to the ring structure of MOX were defined as sites 1 and 2. The groups in sites 1 and 2 were substituted and modified to improve the combined biodegradability of FQs (Figure 2).

**Figure 3:**
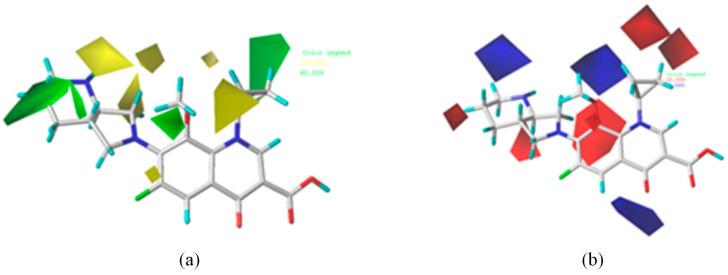
Contour maps of CoMFA model, (**a**) steric field, (**b**) electrostatic field. In the steric field, green contours showed that bulky groups increased the combined biodegradability of FQs, while yellow contours showed that bulky groups decreased the combined biodegradability of FQs. In the electrostatic field, blue contours showed that positive charges increased the combined biodegradability of FQs, and red contours showed that negative charges decreased the combined biodegradability of FQs.

Taking the contour maps of MOX in the comprehension model as an example (Figure 3), the contribution rates of the steric and electrostatic fields were 75.50% and 24.50%, respectively, confirming that space effects and electrostatic interactions affected the combined biodegradability of FQs. Specifically, 1-substituent was surrounded by green, yellow, and red contours, respectively. The color of the contours can be explained as the introduction of smaller, bigger, or negatively charged groups at the site of 1-substituent can improve the combined biodegradability of FQs; 2-substituent was surrounded by green and blue contours, respectively. The color of the contours can be explained as the introduction of bigger or positively charged groups at the site of 2-substituent will improve the combined biodegradability of FQs. Based on this analysis, the substitution of MOX was performed with 10 groups (NO, COOH, CH_3_, C_2_H_5_, C_3_H_7_, C_4_H_9_, C_5_H_11_, C=C, CH_2_NH_2_, and NH_2_) to generate 10 MOX derivatives.

#### 3.3.2. Evaluation of Biodegradability, Environmental Friendliness, and Functionality of MOX Derivatives

Ten MOX derivatives with significantly enhanced combined biodegradability were screened by the comprehension model (Table 4). In the comprehension biodegradation model, the combined biodegradabilities of 10 MOX derivatives were increased by 0.43–25.08% compared with MOX, with Derivative-7 showing the largest increase (25.08%). In the 1GKQ biodegradation model, the single biodegradabilities of 10 MOX derivatives were increased by 6.61–6.82% compared with MOX, with Derivative-3 showing the greatest improvement (6.82%). In the 1OB0 biodegradation model, 10 MOX derivatives’ single biodegradability was increased by 1.52–2.76% compared with MOX, with Derivative-10 showing the greatest improvement (2.76%). In the 5M0K biodegradation model, the single biodegradabilities of 10 MOX derivatives were increased by 6.56–9.57% compared with MOX, with Derivative-7 showing the greatest improvement (9.57%). The above results showed that the binding abilities of all 10 MOX derivatives to the target proteins were increased compared with MOX, indicating that the biodegradabilities of MOX derivatives were improved for both the single thermophilic bacteria and combined thermophilic group. Among them, the combined biodegradability was the most significant.

In addition, based on ensuring that the biodegradabilities of MOX derivatives were all improved, this study used the existing 3D-QSAR models based on photodegradability (log*t*_1/2_) [35], bioconcentration (log*K*_ow_) [36], and the existing H-QSAR model based on genotoxicity (*pLOEC*) [37] of FQs, respectively, to evaluate and predict the environmental friendliness and functionality of the 10 MOX derivatives (Table 5). In the photodegradability, the log*t*_1/2_ values of 10 MOX derivatives were reduced compared to MOX, with Derivative-9 showing the largest reduction (129.47%), indicating that the photodegradation rates of the above 10 MOX derivatives were significantly enhanced. In the bioconcentration, according to the definition of persistent pollutants in the “Stockholm Convention on Persistent Organic Pollutants”, log*K*_ow_ > 5 is defined as the red line of bioconcentration, i.e., chemicals with log*K*_ow_ > 5 will be considered to meet the condition of persistence in bioaccumulation. The evaluation results for the bioconcentration showed that the log*K*_ow_ of all 10 MOX derivatives were less than 5, indicating that their bioaccumulation ability was within the acceptable range, among which, the log*K*_ow_ values of Derivative-1, Derivative-2, Derivative-9, and Derivative-10 were significantly decreased compared with MOX. The decreases were 77.79%, 43.05%, 4.00%, and 52.32%, i.e., their bioconcentration was significantly reduced. In the antibiotic functionality, the *pLOEC* values of all 10 MOX derivatives were reduced compared to MOX (decreased by 0.69–10.05%), with Derivative-1 having the smallest predicted value (7.978), indicating that the genotoxicity of all 10 MOX derivatives mentioned above was increased, i.e., their bactericidal potency was increased to varying degrees.

In summary, the MOX derivatives designed based on the 3D-QSAR models were significantly improved or within acceptable limits in photo degradability (129.47%), bioconcentration (77.79%), and genotoxicity (10.05%) while ensuring enhanced biodegradability, so the subsequent research was conducted around the MOX derivatives as an example.

### 3.4. Regulation of External Environmental Conditions to Enhance the Combined Biodegradation of FQs by the Thermophilic Group in Sewage Sludge Composting Stage

#### 3.4.1. Effectiveness Validation of External Environmental Conditions to Enhance the Combined Biodegradation of FQs by Thermophilic Group

In this study, MD simulation was used to simulate the binding of Derivative-10 and 1GKq&1OB0&5M0K in blank and experimental groups, respectively. Derivative-10 was chosen for the study mainly because of its improved functional properties and environmental friendliness. The improvement in the combined biodegradation of Derivative-10 by the thermophilic group in the sewage sludge composting stage under four scenarios was judged by the binding energy obtained from the different external environmental conditions. Finally, the optimal control scheme of external environmental conditions was selected. Specifically, in scenario 1, the composting temperature was 35 °C and C/N was 25; in scenario 2, the composting temperature was 35 °C and C/N was 35; in scenario 3, the composting temperature was 55 °C and C/N was 25; in scenario 4, the composting temperature was 55 °C and C/N was 35.

**Figure 4:**
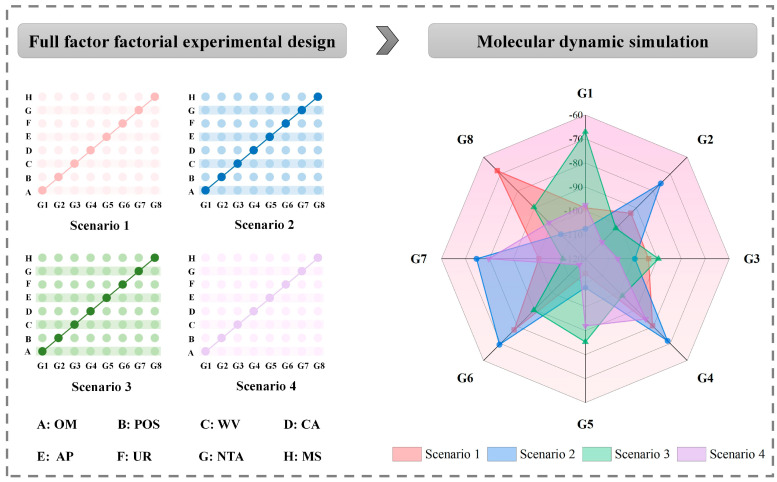
MD simulation results for combined biodegradation of Derivative-10 by the thermophilic group in sewage sludge composting stage under multiple scenarios.

The MD simulation results are shown in Figure 4. In scenario 1, the binding energy of Derivative-10 with 1GKQ&1OB0&5M0K composite system in the blank group (G0) was −102.160 kJ/mol. In the experimental group, the binding energy of Derivative-10 with 1GKQ&1OB0&5M0K composite system in G5 (−113.825 kJ/mol) was improved compared with the blank group, and the combined biodegradation of Derivative-10 by the thermophilic group was increased by 11.42%. It indicated that when the composting temperature was set at 35 °C and C/N was 25, the appropriate addition of AP could effectively improve the interaction between Derivative-10 and 1GKQ&1OB0&5M0K, that is, AP was conducive to the degradation of Derivative-10 by the thermophilic group. In scenario 2, the binding energy of Derivative-10 with the 1GKQ&1OB0&5M0K composite system in the blank group (G0) was −81.934 kJ/mol. In the experimental group, the binding energies of Derivative-10 with 1GKQ&1OB0&5M0K composite system in G1, G3, G5, and G8 were improved compared with the blank group (increased by 21.26–31.74%). Their binding energies were −107.514 kJ/mol, −99.357 kJ/mol, −107.943 kJ/mol, and −105.530 kJ/mol, respectively, indicating that when the compost temperature was set at 35 °C and C/N was 35, the appropriate addition of POS, WV, AP, and MS can effectively enhance the interaction between Derivative-10 and 1GKQ&1OB0&5M0K, that is, POS, WV, AP, and MS were conducive to the degradation of Derivative-10 by the thermophilic group. In scenario 3, the binding energy of Derivative-10 with the 1GKQ&1OB0&5M0K composite system in the blank group (G0) was −110.642 kJ/mol. In the experimental group, the binding energies of Derivative-10 with the 1GKQ&1OB0&5M0K composite system in eight experimental groups were lower than that in the blank group, indicating that when the composting temperature was set at 55 °C and C/N was 25, POS, OM and six exogenous additives could not improve the degradation ability of Derivative-10 by the thermophilic group. In scenario 4, the binding energy of Derivative-10 with 1GKQ&1OB0&5M0K composite system in the blank group (G0) was −100.372 kJ/mol. In the experimental group, the binding energies of Derivative-10 with 1GKQ&1OB0&5M0K composite system in G2, G3 and G6 were improved compared with the blank group (increased by 6.11–16.01%). Their binding energies were −110.14 kJ/mol, −106.506 kJ/mol, and −116.439 kJ/mol, respectively, indicating that when the compost temperature was set at 55 °C and C/N was 35, the appropriate addition of POS, WV, and UR can effectively enhance the interaction between Derivative-10 and 1GKQ&1OB0&5M0K, that is, POS, WV, and UR were conducive to the degradation of Derivative-10 by the thermophilic group. Based on the above analysis results, it can be seen that the key influencing factors that effectively stimulate the degradation of Derivative-10 by thermophilic bacteria in different sludge aerobic composting environments differed significantly. Also, the effectiveness of key influencing factors in stimulating the degradation of Derivative-10 by thermophilic flora was inconsistent, and this conclusion is consistent with the findings from Yang et al. who found that various additives also differed in different composting raw material ratios [38]. For example, the complex system with Derivative-10 and 1GKQ&1OB0&5M0K had the strongest binding ability in the blank group of scenario 3, indicating that the thermophilic group already had high biological activity in this composting environment, and it can effectively degrade FQs. We speculated that the addition of key influence factors may break the existing equilibrium and eventually lead to a decrease in the degradation ability of the thermophilic group to Derivative-10. On the contrary, the complex system with Derivative-10 and 1GKQ&1OB0&5M0K had the weakest binding ability in the blank group of scenario 2, indicating that the thermophilic group had lower biological activity in this composting environment, and its ability to degrade FQs was weak. We speculated that when appropriate key influence factors were added to this composting environment, the bioactivity of the thermophilic group would be stimulated appropriately, and the degradation ability of the thermophilic group to Derivative-10 would be finally improved. In conclusion, it is of great significance to clarify the effect of different composting temperatures and C/N on soil biological properties, which is of great significance for the efficient degradation of FQs by thermophilic bacteria in the sewage sludge composting stage.

#### 3.4.2. Identification of Interactions between the External Environmental Conditions for Enhancing the Combined Biodegradation of FQs by Thermophilic Group

Since the levels of each factor in the full factorial experiment were controllable, and all the factors were fixed factors, the fixed-effects model was used to analyze the variance and effect (significance level at *p* = 0.05) of the main effect and the second-order interaction effect of the external environmental conditions to enhance the combined biodegradation of FQs by thermophilic group [39]. In scenario 2, the results for the main effect showed that the effect estimates of POS, WV, AP, and MS were 2.283, −5.908, 10.929, and 5.792, respectively, among which the estimated effects of POS, AP, and MS were positive, and the estimated effect of WV was negative, indicating that POS, AP, and MS had to promote effects on the combined biodegradability of FQs by thermophilic group, while WV had an inhibitory effect. In the second-order interactive effects, the effect estimates of POS&WV and POS&AP were −0.162 and −15.048, indicating that the above two regulatory schemes had significant inhibitory effects on the combined biodegradability of FQs by thermophilic group, that is, the simultaneous application of POS and WR, and POS and AP to the sewage sludge composting environment can inhibit the degradation of FQs by thermophilic bacteria; the effect estimate of POS&MS was 6.134, indicating that this regulatory scheme had a significant synergistic effect on the combined biodegradability of FQs by thermophilic group, that is, the simultaneous application of POS and MS to the sewage sludge composting environment can promote the degradation of FQs by thermophilic bacteria. In scenario 4, the results for the main effect showed that the effect estimates of OM, WV, and AP were −7.01, −15.27, and 13.43, respectively, among which the estimated effect of AP was positive, and the estimated effects of OM and WV were negative, indicating that AP had a promoting effect on the combined biodegradability of FQs by thermophilic group, while OM and WV had inhibitory effects. In the second-order interactive effects, the effect estimate of OM&WV was −20.68, indicating that this regulatory scheme had a significant inhibitory effect on the combined biodegradability of FQs by the thermophilic group, that is, the simultaneous application of OM and WV to the sewage sludge composting environment can inhibit the degradation of FQs by thermophilic bacteria; the effect estimates of OM&AP and WV&AP were 19.26 and 15.82, indicating that the above two regulatory schemes had significant synergistic effects on the combined biodegradability of FQs by thermophilic group, that is, the simultaneous application of OM and AP, and WV and AP to the sewage sludge composting environment can promote the degradation of FQs by thermophilic bacteria. In the third-order interactive effects, the effect estimate of OM&WV&AP was 10.90, indicating that this regulatory scheme had a significant synergistic effect on the combined biodegradability of FQs by the thermophilic group, that is, the simultaneous application of OM, WV, and AP to the sewage sludge composting environment can promote the degradation of FQs by thermophilic bacteria. Compared with the existing research results, the mixed-use of sulfur powder and monoammonium phosphate can reduce the pH of the material and decrease the conversion of NH_4_^+^-N to ammonia gas, thereby improving the degradation ability of microbial flora to FQs [40]. Tao et al. mixed different proportions of AP and 1% CA into pig manure and straw for aerobic composting and found that the fruit acid in AP can reduce nitrogen loss [41]. Although the results for the above research did not directly discuss the combined effect of the various additives involved in this study in promoting the biodegradation of FQs, it indirectly verified that there was a certain interaction relationship between the different additives proposed in this paper, and AP has a positive effect on promoting the biodegradation of FQs.

### 3.5. Regulatory Measures to Enhance the Ability of FQs Antibiotics to Inhibit Expression of Bacterial Resistance in Agricultural Soils

Because it contains a large amount of OM and nutrients required by plants and has strong farmland fertility [42], the agricultural application of sludge is considered to be an active and effective sludge disposal method [43]. However, incompletely treated contaminants such as ARGs are present in sludge that can be used for agricultural application, and ARGs will enter the soil environment with the agricultural application of sludge and gradually develop bacterial resistance [22]. This paper screens field application options based on molecular docking, sampling methods, and MD simulation to screen field application options that effectively enhance the resistance of MOX derivatives to bacterial resistance in agricultural soil environments.

#### 3.5.1. Characterization of the Ability of FQs Antibiotics to Inhibit the Expression of Bacterial Resistance

**Figure 5:**
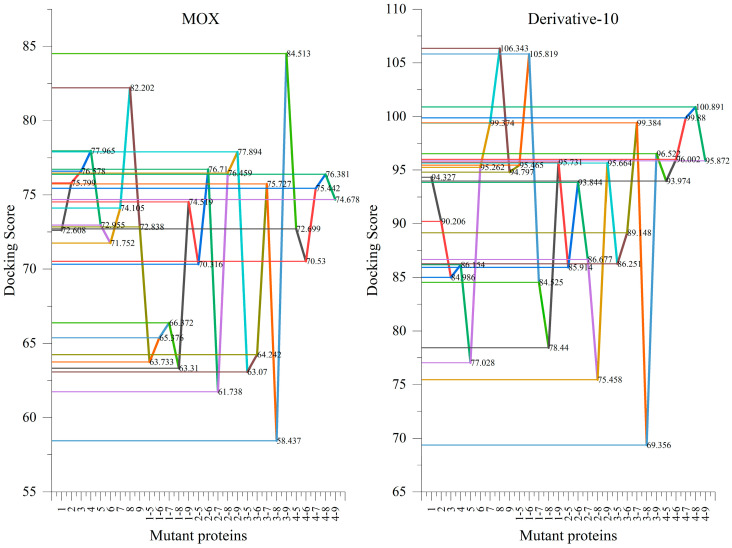
Docking results for MOX and Derivative-10 with all mutant proteins of *Escherichia coli* DNA gyrase.

In the bactericidal mechanism of FQ antibiotics, FQ antibiotics bind to DNA gyrase in Gram-negative bacteria, inhibiting DNA gyrase’s participation in the process of bacterial binary division, to achieve its bactericidal purpose [16]. In addition, DNA gyrase is both a participant and an outcome of the self-replication process in Gram-negative bacteria, so Gram-negative bacteria carrying FQ antibiotic-resistant genes will naturally produce FQ antibiotic-resistant DNA gyrase mutant proteins while they are self-replicating, guided by the antibiotic-resistant genes, thereby inhibiting the effective binding of FQ antibiotics to them, and thus affecting the bactericidal effect of FQ antibiotics. This in turn affects the bactericidal efficacy of FQs antibiotics by inhibiting their effective binding, leading to bacterial resistance. In this paper, based on the study by Ren et al., 29 *Escherichia coli* DNA gyrase mutant proteins were selected from their study as representatives of *Escherichia coli* bacterial resistance expression [25]. The target group (later referred to as the 2–8 mutant protein) was selected as the group with the least effective molecular docking between Derivative-10 and each of the mutant proteins compared to the MOX molecular docking (Figure 5), and the target group was then subjected to MD simulation of the effect of each field application regimen on the ability of MOX and Derivative-10 to inhibit bacterial resistance expression in agricultural soil.

#### 3.5.2. A Survey on Background Values of Nutrients in Agricultural Soils Based on the Sampling Method

To effectively stimulate the agricultural soil background, the data were sourced from the National Soil Information Service Platform of China (http://www.soilinfo.cn, accessed on 1 February 2022). The sampling survey method was employed; the content of the nutrient elements, C, N, and P in the soil of the agricultural area were obtained to obtain the basic simulation number of C, N, and P contents in the soil of the agricultural area. Among them, the sampling survey method refers to a survey method that selects some units from all the units of the research object for investigation and analysis and uses the quantitative characteristics of these units to infer the overall quantitative characteristics [44]. In this paper, a total of 12 sample points were selected for data collection based on the sampling survey method and the data of the National Soil Information Server (Table 6).

In this paper, the background value of nutrient elements in agricultural soil was investigated, and the number of nutrient elements added to agricultural soil was determined. Based on this, the MD simulation of FQ antibiotics inhibiting bacterial resistance expression in agricultural soil before and after MOX molecular modification was carried out. The main purpose was to determine whether the ability of MOX to inhibit bacterial resistance expression in agricultural soil before and after modification is improved through MD simulation and to identify and screen field measures that are beneficial to FQ antibiotics in agricultural soil to inhibit bacterial resistance expression. Among them, field measures refer to improving the physical and chemical properties and cultivation capacity of soil in agricultural areas through different physical, chemical, and biological means [45]. Specifically, straw returning and organic fertilizer application are widely advocated as organic green measures and methods, which can not only consume waste OM energy but also enhance soil fertility and restore farming ability [46]. Therefore, the field measures selected in this paper include returning straw to the field, increasing the application of organic fertilizers, and plowing. The representative substances are p-coumarol [47], fulvic acid [48], and oxygen.

#### 3.5.3. Validation of Field Measures to Strengthen FQs Antibiotics to Inhibit Bacterial Resistance Expression

First, the MD simulation experiments of the ability of MOX and Derivative-10 to inhibit the expression of bacterial resistance in the soil of agricultural areas under the blank background were carried out (Table 7). The simulation results showed that compared with MOX, the binding ability of Derivative-10 to 2–8 mutant protein was significantly improved (48.87%). It shows that the modified Derivative-10 based on the QSAR model has a better ability to inhibit the expression of bacterial drug resistance in farmland soil than MOX.

After, the binding ability of Derivative-10 to 2–8 mutant protein in the soil of the agricultural area under a blank background was investigated. This paper further simulated the improvement in the ability of Derivative-10 to inhibit the expression of bacterial resistance under different field measures (Table 7). Soil simulation results in the agricultural zone under different field measure application schemes showed that in the experimental group with single field measure application, the plowing measure was superior to the straw return and organic fertilizer application measures. The average binding energy of Derivative-10 and 2–8 mutant protein in the experimental group with organic fertilizer application in the agricultural zone was −93.266 kJ/mol, which was 26.71% higher than that of the single organic fertilizer application. The average binding energy of Derivative-10 and 2–8 mutant protein in the experimental group in the agricultural zone with the straw-returning measure was −90.919 kJ/mol, and the binding capacity was increased by 23.52% compared with the single straw-returning measure. The average binding energy of Derivative-10 and the 2–8 mutant protein in the experimental group in the agricultural zone with plowing was −92.778 kJ/mol, with a 26.04% increase in binding capacity compared to the single plowing measure. The results for the combination of the different field measures were greater than those for the single field measure, i.e., the combination of the field measures was more effective than the single field measure in enhancing the ability of FQ antibiotics to inhibit the expression of bacterial resistance. In summary, the ability of Derivative-10 to inhibit bacterial resistance expression of FQ antibiotics in a blank background was enhanced by the single and combined application of all three field measures, with the greatest increase (41.31%) in the inhibition of bacterial resistance expression of Derivative-10 in agricultural areas with the simultaneous application of three field measures: straw return, organic fertilizer application, and plowing.

## 4. Conclusions

In this study, auxiliary techniques for the entropy weight method, the index normalization method, and full factor factorial experimental design were developed. The biodegradation systems of FQs under single thermophilic bacteria and combined thermophilic group were optimized from both the molecular design with improved biodegradation and the control of external environmental conditions by integrating the 3D-QSAR models, molecule/protein–protein docking, and MD simulation. We achieved the goal of improving the environmental friendliness and functional properties of FQs under multiple systems and multiple scenarios. Finally, the ideal regulation scheme of external environmental conditions that can effectively improve the biodegradability of FQs, and the application scheme of field measures to effectively enhance the antibacterial resistance of FQ derivative molecules in the soil environment after sludge land use were screened out. Compared with conventional studies, a coupled study of molecular modification, regulation of external environmental conditions, and resistance to bacterial drug resistance behavior was used to provide theoretical support for improving the biodegradability of FQs and mitigating the potential risks they may pose to the environment and humans through forms such as food chain transmission.

## Figures and Tables

**Table 1:** Experimental table of external environmental conditions to improve the combined biodegradability of thermophilic bacteria of FQ derivatives in sewage sludge composting stage.

Scenarios	Group	OM	POS	WV	CA	AP	UR	NTA	MS
**Scenarios 1–4**	**0**	0	0	0	0	0	0	0	0
	**1**	1	0	0	0	0	0	0	0
	**2**	0	1	0	0	0	0	0	0
	**3**	0	0	1	0	0	0	0	0
	**4**	0	0	0	1	0	0	0	0
	**5**	0	0	0	0	1	0	0	0
	**6**	0	0	0	0	0	1	0	0
	**7**	0	0	0	0	0	0	1	0
	**8**	0	0	0	0	0	0	0	1

Note: wood vinegar (WV), citric acid (CA), apple pomace (AP), urethane residue (UR), nitrilotriacetic acid (NTA), and medical stone (MS).

**Table 2:** Biodegradability levels of FQs at different target receptors (characterized by the Libdock Scores, Å).

No.	LibDock Scores (Å)			No.	LibDock Scores (Å)		
	1GKQ	1OB0	5M0K		1GKQ	1OB0	5M0K
DIF	84.12	75.48	140.10	GRE	112.04	96.52	131.73
ENR	100.78	86.76	134.64	ORB	109.02	73.32	123.93
NOR	104.68	72.91	112.09	SIT	113.51	44.62	116.03
LOM	101.81	77.37	102.15	TEM	101.77	66.88	128.92
OFL	98.41	78.28	116.92	D1	97.29	65.18	117.57
PEF	99.23	79.78	116.54	D12	103.11	62.64	121.15
FLE	99.72	79.14	119.69	D13	110.35	72.08	140.83
CIP	104.38	70.57	121.27	D14	108.14	72.45	126.07
BAL	93.22	70.74	112.77	D16	111.53	70.42	118.88
MAR	95.34	71.23	112.43	D28	105.93	68.43	119.98
PIP	98.32	80.33	115.43	D29	106.31	72.58	93.84
CIN	82.01	77.73	106.75	D32	109.95	66.48	123.98
ENO	105.20	69.22	116.45	D36	108.31	71.45	111.83
DAN	113.74	74.73	117.32	D37	109.31	77.10	124.29
GAT	93.02	54.82	104.21	F1	101.62	83.50	118.65
LEV	98.41	78.28	116.92	F2	111.46	77.43	111.20
RUF	82.48	77.59	102.54	F3	61.76	72.02	56.16
PAZ	95.01	79.25	123.49	F4	91.93	76.03	91.83
NAD	108.43	81.41	112.09	F5	99.71	65.73	113.26
MOX	83.16	69.91	83.47	F6	113.41	81.72	105.99
SPA	114.87	72.45	121.10	Gat-29	116.29	72.32	130.15
SAR	88.81	75.82	135.60	Gat-30	117.05	77.81	129.55
AMI	97.24	73.04	105.85	Gat-31	91.29	63.80	117.09
BES	82.54	59.00	119.14	Gat-33	106.93	80.69	125.73
CLI	109.08	69.81	115.70				

**Table 3:** Calculation of the comprehension value (CV) of combined biodegradation of 49 FQs by thermophilic group (1GKQ&1OB0&5M0K).

No.	CV	No.	CV	No.	CV	No.	CV
1	5.857	14	6.205	27	6.185	F1	6.154
2	6.792	15	4.129	28	4.882	F2	6.027
3	5.605	16	5.752	29	5.800	F3	2.050
4	5.351	17	4.630	D1	5.177	F4	4.571
5	5.752	18	5.881	D12	5.413	F5	5.149
6	5.834	19	6.108	D13	6.747	F6	6.110
7	5.931	20	3.696	D14	6.185	Gat-29	6.629
8	5.801	21	6.278	D16	5.990	Gat-30	6.871
9	5.095	22	5.902	D28	5.727	Gat-31	4.872
10	5.186	23	5.116	D29	5.045	Gat-33	6.475
11	5.785	24	4.401	D32	5.933		
12	4.758	25	5.764	D36	5.675		
13	5.615	26	7.541	D37	6.367		

**Table 4:** The single and comprehensive biodegradation of MOX derivatives.

Compounds	Substituent Groups	Predicted CV	Change Range (%)	Predicted 1GKQ	Change Range (%)	Predicted 1OB0	Change Range (%)	Predicted 5M0K	Change Range (%)
Moxifloxacin	-	**3.696**	-	**1.920**	-	**1.845**	-	**1.922**	-
Derivative-1	1-NO	**3.823**	3.44	**2.049**	6.72	**1.873**	1.52	**2.062**	7.28
Derivative-2	1-COOH	**3.712**	0.43	**2.048**	6.67	**1.880**	1.90	**2.066**	7.49
Derivative-3	2-CH_3_	**4.079**	10.36	**2.051**	6.82	**1.891**	2.49	**2.058**	7.08
Derivative-4	2-C_2_H_5_	**4.315**	16.75	**2.049**	6.72	**1.889**	2.38	**2.073**	7.86
Derivative-5	2-C_3_H_7_	**4.592**	24.24	**2.049**	6.72	**1.887**	2.28	**2.093**	8.90
Derivative-6	2-C_4_H_9_	**4.592**	24.24	**2.049**	6.72	**1.885**	2.17	**2.100**	9.26
Derivative-7	2-C_5_H_11_	**4.623**	25.08	**2.049**	6.72	**1.883**	2.06	**2.106**	9.57
Derivative-8	2-C=C	**4.144**	12.12	**2.050**	6.77	**1.885**	2.17	**2.070**	7.70
Derivative-9	2-CH_2_NH_2_	**4.088**	10.61	**2.050**	6.77	**1.890**	2.44	**2.064**	7.39
Derivative-10	2-NH_2_	**4.454**	20.51	**2.047**	6.61	**1.896**	2.76	**2.048**	6.56

**Table 5:** Environmental friendliness and functional evaluation of MOX derivatives.

No.	Genotoxicity		Bioaccumulation		Photodegradability	
	Predicted	Change Range (%)	Predicted	Change Range (%)	Predicted	Change Range (%)
MOX	8.869		0.950		1.975	
Derivative-1	7.978	−10.05	0.211	−77.79	0.965	−51.14
Derivative-2	8.163	−7.96	0.541	−43.05	0.921	−53.37
Derivative-3	8.389	−5.41	1.147	20.74	1.07	−45.82
Derivative-4	8.343	−5.93	1.258	32.42	1.094	−44.61
Derivative-5	8.437	−4.87	1.497	57.58	1.117	−43.44
Derivative-6	8.537	−3.74	1.547	62.84	1.153	−41.62
Derivative-7	8.467	−4.53	1.643	72.95	1.156	−41.47
Derivative-8	8.373	−5.59	1.102	16.00	1.068	−45.92
Derivative-9	8.549	−3.61	0.912	−4.00	−0.582	−129.47
Derivative-10	8.808	−0.69	0.453	−52.32	−0.219	−111.09

**Table 6:** Investigation on background values of nutrient elements in agricultural soil based on the sampling method.

Area	No.	Coordinate		Nutrient Content (g/kg)		
		E (°)	N (°)	C	N	P
I	1	125.60	50.15	1.33	0.85	2.17
	2	125.62	48.46	0.87	0.56	1.27
	3	125.56	46.64	0.43	0.24	0.84
	4	125.45	45.23	0.68	0.58	0.99
II	5	120.20	40.41	0.96	0.53	1.23
	6	122.95	42.00	0.76	0.49	1.14
	7	124.79	41.33	0.37	0.24	0.57
	8	121.44	38.91	0.96	0.53	1.23
III	9	125.97	41.94	2.13	0.49	1.14
	10	120.07	43.63	1.42	0.88	1.36
	11	127.55	42.82	1.23	0.72	0.99
	12	130.71	44.47	2.13	1.15	2.17
Average value				1.11	0.61	1.26
Addition of Nutrient Elements				2	1	2

Note: In this paper, the northeast area is selected as the research background according to the agricultural zoning rules. Its zoning attributes are the eastern humid, semi-humid, iron-rich, leaching, alumino–siliceous soil area, and the soil zone to which it belongs is the dark brown soil chernozem zone.

**Table 7:** Effects of Derivative-10 on the expression of bacterial drug resistance under different field measures.

FQs	Nutrient Elements			Field Measures			Binding Energy	Change Range
	C	N	P	Organic Fertilizer Application	Straw Returning	Plowing		
MOX	2	1	2	-	-	-	−49.445	-
Derivative-10				-	-	-	−73.608	48.87%
				0	0	0	−73.608	-
				1	0	0	−85.565	16.24%
				0	1	0	−78.515	6.67%
				0	0	1	−93.523	27.06%
				1	1	0	−95.529	29.78%
				1	0	1	−87.956	19.49%
				0	1	1	−85.619	16.32%
				1	1	1	−104.013	41.31%

## Data Availability

The data presented in this study are available contained within the article.
